# Supplementary material for: Nonspecific Binding of a Putative S-Layer Protein to Plant Cell Wall Polysaccharides—Implication for Growth Competence of Lactobacillus brevis in the Gut Microbiota
Source: Int J Mol Sci. 2025 Nov 30;26(23):11612. doi: 10.3390/ijms262311612 (PMC12691797; doi:10.3390/ijms262311612)
Supplement: Supplementary file 1 [file ijms-26-11612-s001.zip › ijms-3940254-supplementary.pdf]

International Journal of
Molecular Sciences

MDPI

Supplementary materials

Nonspecific binding of a putative S-layer protein to plant cell wall polysaccharides - implication for growth competence of *Lactobacillus brevis* in the gut microbiota

Zhenzhen Hao [1], Wenjing Zhang [1,2], Jianzhong Ge [1], Daoxin Yang [1], Kairui Guo [1], Yuan Wang [1], Huiying Luo [1], Huoqing Huang [1,*] and Xiaoyun Su [1,*]

Supplementary Table S1. Primers used in this study

Primer	Sequence (5′–3′)	Usage
PLb634F	CTGGTGCCGCGCGGCAGCGATGTTCAGCAATCTACT	Cloning of *Lb634* into pET-28a(+)
PLb634R	GGTGGTGGTGCTCGAGTTTAATTTTCTTGCTTCC-TACGTT	Cloning of *Lb634* into pET-28a(+)
PLb636F	TGGTGCCGCGCGGCAGCAATTCAGGGTCATTGTC	Cloning of *Lb636* into pET-28a(+)
PLb636R	GGTGGTGGTGCTCGAGTTTATTGCGGCGTATCAG-TCAGTAA	Cloning of *Lb636* into pET-28a(+)
PLb1145F	CTGGTGCCGCGCGGCAGCAAGTCATACGCTACTGCT	Cloning of *Lb1145* intopET-28a(+)
PLb1145R	GGTGGTGGTGCTCGAGTTTAAGCAATCCAGC-TACTGTCAG	Cloning of *Lb1145* into pET-28a(+)
PLb1181F	TGGTGCCGCGCGGCAGCAAAGAGTACCAAGCGGC	Cloning of *Lb1181* into pET-28a(+)
PLb1181R	GGTGGTGGTGCTCGAGTTTATTGCAGATATTGTTT-GGCGT	Cloning of *Lb1181* into pET-28a(+)
PLb1345F	CCGCGCGGCAGCCATATGATGGTT-GCTAAACAACAC	Cloning of *Lb1345* into pET-28a(+)
PLb1345R	GGTGGTGGTGCTCGAGTTTATGCGTTTT-GTTCAATTAACTTGCG	Cloning of *Lb1345* into pET-28a(+)
PLb2328F	TGGTGCCGCGCGGCAGCAAGAGTACCAATAAGAC	Cloning of *Lb2328* into pET-28a(+)
PLb2328R	GGTGGTGGTGCTCGAGTTCATTTACCAAC-GGTCCGGTAA	Cloning of *Lb2328* into pET-28a(+)
PLb2458F	TGGTGCCGCGCGGCAGCGCTGGCACCAAAGTTGC	Cloning of *Lb2458* into pET-28a(+)
PLb2458R	GTGGTGGTGGTGGTGCTCGAGTTTAGTAAGTT-GAAACGTAACCCTTATT	Cloning of *Lb2458* into pET-28a(+)
PLb2554F	TGGTGCCGCGCGGCAGCAGTATTACGACATCTGG	Cloning of *Lb2554* into pET-28a(+)
PLb2554R	GGTGGTGGTGCTCGAGTTTAGTGAACAC-CATCTACCAGGG	Cloning of *Lb2554* into pET-28a(+)
PLb1145F$_0$	CTGGTGCCGCGCGGCAGCAAGTCATACGC-TACTGCTGG	Cloning of *Lb1145* and *DsRed* into pET-28a(+)
PLb1145R$_0$	TGTTGTCCATAGCAATCCAGCTACTGTCAGATGAA-GATGCTGAAGGAGC	Cloning of *Lb1145* and *DsRed* into pET-28a(+)
PDsRedF$_0$	CTGGATTGCTATGGACAACACCGAGGAC-GTCATCAAGGA	Cloning of *Lb1145* and *DsRed* into pET-28a(+)
PDsRedF$_0$	TGGTGGTGCTCGAGTTTACTGGGAGCCGGAG-	Cloning of *Lb1145* and *DsRed* into pET-28a(+)

	TGGCGGGCC	
P1145TM1F	CCTGGTGCCGCGCGGCAGCAAGTCATACGC-TACTGCTG	Cloning of *Lb1145* domain1 into pET-28a(+)
P1145TM1R	GGTGGTGGTGCTCGAGTTTACGTGTTAGCAGACTT-GATACCACCAGCAA	Cloning of *Lb1145* domain1 into pET-28a(+)
P1145TM2F	CTGGTGCCGCGCGGCAGCACGACTACTGC-TACTACGC	Cloning of *Lb1145* domain2 into pET-28a(+)
P1145TM2R	GGTGGTGGTGCTCGAGTTTATGCACCAGCAAC-GTAACCCTTACCTGCGT	Cloning of *Lb1145* domain2 into pET-28a(+)
P1145TM1F1	CCTGGTGCCGCGCGGCAGCAAGTCATACGC-TACTGCTG	Cloning of *Lb1145* domain1 and *DsRed* into pET-28a(+)
P1145TM1R1	TGTTGTCCATCGGATCGCTACGTTCCAGCG-GATCACCCGTGTTAGCAGACT	Cloning of *Lb1145* domain1 and *DsRed* into pET-28a(+)
PDsRedF1	TAGCGATCCGATGGACAACACCGAGGAC-GTCATCAAGGAGTTCATGCAG	Cloning of *Lb1145* domain1 and *DsRed* into pET-28a(+)
PDsRedR1	GGTGGTGGTGCTCGAGTTTACTGGGAGCCGGAG-TGGCGG	Cloning of *Lb1145* domain1 and *DsRed* into pET-28a(+)
P1145TM2F	TGGTGCCGCGCGGCAGCACGACTACTGCTACTAC	Cloning of *Lb1145* domain2 and *DsRed* into pET-28a(+)
P1145TM2R	TGTTGTCCATCGGATCGCTACGTTCCAGCG-GATCACCTGCACCAGCAACGT	Cloning of *Lb1145* domain2 and *DsRed* into pET-28a(+)
PDsRedF2	TAGCGATCCGATGGACAACACCGAGGAC-GTCATCAAGGAGTTCATGCAG	Cloning of *Lb1145* domain2 and *DsRed* into pET-28a(+)
PDsRedR2	GGTGGTGGTGCTCGAGTTTACTGGGAGCCGGAG-TGGCGG	Cloning of *Lb1145* domain2 and *DsRed* into pET-28a(+)
P1145TM3F	CTGGTGCCGCGCGGCAGCACGACTCAAGACCTT-GGTG	Cloning of *Lb1145* domain3 into pET-28a(+)
P1145TM3R	GGTGGTGGTGCTCGAGTTTAAGCAGCGGTTAC-GTCAACATATACAGTGT	Cloning of *Lb1145* domain3 into pET-28a(+)
P1145TM4F	CCTGGTGCCGCGCGGCAGCGC-TACTTCAAAGGTACAAT	Cloning of *Lb1145* domain4 into pET-28a(+)
P1145TM4R	GGTGGTGGTGCTCGAGTTTAAGCAATCCAGC-TACTGTCAGATGAAGATG	Cloning of *Lb1145* domain4 into pET-28a(+)
P1145TM5F	TGGTGCCGCGCGGCAGCAAGTCATACGCTACTGCTG	Cloning of *Lb1145* domain1+2 into pET-28a(+)
P1145TM5R	GGTGGTGGTGCTCGAGTTTACAATGAAAGACCAC-CAAGGTCTTGAGTCG	Cloning of *Lb1145* domain1+2 into pET-28a(+)
PDsRedF	TGGTGCCGCGCGGCAGCATGGACAACACCGAGGA	Cloning of *DsRed* into pET-28a(+)
PDsRedR	GGTGGTGCTCGAGTTTACTGGGAGCCGGAGTGGC	Cloning of *DsRed* into pET-28a(+)
PegfpF	TGGTGCCGCGCGGCAGCATGGTGTCCAAAGGTGA	Cloning of *egfp* into pET-28a(+)
PegfpR	GGTGGTGGTGGTGCTCGAGTTTTATA-CAATTCATCCATACCT	Cloning of *egfp* into pET-28a(+)
PLb630F_0	TGGTGCCGCGCGGCAGCGACTCAACCCAAAAGAC	Cloning of *Lb630* and *egfp* into pET-28a(+)
PLb630R_0	GGGTAATACGCCGGAAGGTGGTGGTGGTAGCATG	Cloning of *Lb630* and *egfp* into pET-28a(+)
PegfpF_0	GGAAGGTGGTGGTGGTAG-CATGGTGTCCAAAGGTGAAG	Cloning of *Lb630* and *egfp* into pET-28a(+)
PegfpR_0	GGTGGTGGTGGTGCTCGAGTTTTATA-CAATTCATCCATACCTAACGTGA	Cloning of *Lb630* and *egfp* into pET-28a(+)

Supplementary Table S2. A search of high pI proteins in reported cell surface proteins in representative gut commensal bacteria

Strain	Accession number	Predicted function	Number of domains	Isoelectric point (pI)							Ref.
				Full length	Domains						
					1	2	3	4	5	6	
**Gram positive**											
Bifidobacterium animalis subsp. *lactis* Bl12	AGO52940.1	Putative S-layer domain protein	1	9.34	9.34	-	-	-	-	-	[72]
Bifidobacterium choerinum LMG10510	KFI54244.1	Putative S-layer domain protein	3	9.18	10.45	8.54	7.58	-	-	-	[72]
Bifidobacterium gallicum LMG11596	KFI59699.1	Putative S-layer domain protein	1	9.58	9.58	-	-	-	-	-	[72]
Bifidobacterium pseudolongum LMG 11569	KFI75058.1	Putative S-layer domain protein	1	10.02	10.02	-	-	-	-	-	[72]
Bifidobacterium pseudolongum LMG 11569	KFI75530.1	Putative S-layer domain protein	1	9.96	9.96	-	-	-	-	-	[72]
Lactobacillus acidophilus CICC 6074	WP_011254065.1	Pore-forming S-layer protein SlpA	3	9.59	4.85	7.87	9.92	-	-	-	[66]
Lactobacillus acidophilus NCFM	AAV43395.1	Putative serine protease	5	9.54	9.94	9.7	4.85	6.28	9.71	-	[73]
Lactobacillus acidophilus NCFM	AAV42570.1	Hypothetical protein	2	9.24	9.35	8.54	-	-	-	-	[73]
Lactobacillus acidophilus NCFM	AAV43384.1	Aminopeptidase	2	9.47	9.71	8.93	-	-	-	-	[73]
Lactobacillus acidophilus NCFM	AAV43059.1	Hypothetical protein	1	9.90	9.90	-	-	-	-	-	[73]
Lactobacillus acidophilus NCFM	AAV42118.1	Cell separation protein CdpA	5	9.80	10.39	9.49	9.25	9.06	5.39	-	[73]
Lactobacillus acidophilus NCFM	AAV43130.1	Oligopeptide ABC trasporter substrate binding protein	1	9.55	9.55	-	-	-	-	-	[73]
Lactobacillus acidophilus NCFM	AAV42013.1	Putative glutamine ABC transporter	1	9.80	9.80	-	-	-	-	-	[73]
Lactobacillus acidophilus NCFM	AAV43328.1	Penicillin-binding protein	1	9.52	9.52	-	-	-	-	-	[73]
Lactobacillus acidophilus NCFM	AAV42669.1	Penicillin-binding protein	2	9.74	9.74	9.61	-	-	-	-	[73]
Lactobacillus acidophilus NCFM	AAV43251.1	Hypothetical protein	3	10.11	9.46	4.51	10.38	-	-	-	[73]
Lactobacillus acidophilus NCFM	AAV42719.1	Penicillin-binding protein	1	10.24	10.24	-	-	-	-	-	[73]
Lactobacillus acidophilus NCFM	AAV43715.1	Lysin	2	10.28	9.49	10.79	-	-	-	-	[73]
Lactobacillus acidophi-	AAV42117.1	Hypothetical protein	2	10.23	10.21	10.16	-	-	-	-	[73]

lus NCFM											
Lactobacillus acidophilus NCFM	AAV43358.1	Hypothetical protein	1	9.93	9.93	-	-	-	-	-	[73]
Lactobacillus acidophilus NCFM	AAV43502.1	Putative membrane protein	1	9.66	9.66	-	-	-	-	-	[73]
Lactobacillus acidophilus NCFM	AAV42723.1	Hypothetical protein	1	9.66	9.66	-	-	-	-	-	[73]
Lactobacillus acidophilus NCFM	AAV43551.1	Hypothetical protein	1	9.62	9.62	-	-	-	-	-	[73]
Lactobacillus acidophilus NCFM	AAV43555.1	Putative glycosidase	1	9.94	9.94	-	-	-	-	-	[73]
Lactobacillus acidophilus NCFM	AAV41921.1	Putative alkylphosphonate ABC transporter	1	9.65	9.65	-	-	-	-	-	[73]
Lactobacillus acidophilus NCFM	AAV43416.1	Putative cell surface protein	1	9.94	9.94	-	-	-	-	-	[73]
Lactobacillus acidophilus NCFM	AAV42860.1	Hypothetical protein	2	10.06	9.41	10.54	-	-	-	-	[73]
Lactobacillus acidophilus NCFM	AAV42037.1	Hypothetical protein	2	9.64	9.24	9.77	-	-	-	-	[73]
Lactobacillus acidophilus NCFM	AAV43418.1	D-alanyl-D-alanine carboxypeptidase	2	9.70	9.38	10.10	-	-	-	-	[73]
Lactobacillus acidophilus NCFM	AAV42035.1	Glutamine ABC transporter permease protein	1	9.78	9.78	-	-	-	-	-	[73]
Lactobacillus acidophilus NCFM	AAV41945.1	Hypothetical protein	1	10.30	10.30	-	-	-	-	-	[73]
Lactobacillus acidophilus NCFM	AAV42859.1	Secreted protein	1	9.89	9.89	-	-	-	-	-	[73]
Lactobacillus acidophilus NCFM	AAV41985.1	Putative heat shock related serine protease	2	9.42	8.57	9.99	-	-	-	-	[73]
Lactobacillus acidophilus NCFM	AAV41950.1	Hypothetical protein	1	10.21	10.21	-	-	-	-	-	[73]
Lactobacillus acidophilus NCFM	Q5FM22.1	RplA-50S ribosomal protein L1	1	8.89	8.89	-	-	-	-	-	[73]
Lactobacillus acidophilus NCFM	Q5FM84.1	30S ribosomal protein S3	2	10.13	9.60	10.70	-	-	-	-	[73]
Lactobacillus acidophilus NCFM	Q5FM89.1	50S ribosomal protein L4	1	10.28	10.28	-	-	-	-	-	[73]
Lactobacillus acidophilus NCFM	AAV43652.1	Putative aggregation promoting protein	1	10.05	10.05	-	-	-	-	-	[73]
Lactobacillus acidophilus NCFM	AAV42877.1	Putative surface layer protein	3	9.74	10.09	9.81	4.93	-	-	-	[73]
Lactobacillus acidophilus NCFM	AAV42394.2	S-layer protein (SlpX)	5	9.55	5.73	5.14	10.00	6.76	10.37	-	[73]
Lactobacillus acidophilus NCFM	AAV42856.1	Penicillin-binding protein	1	9.63	9.63	-	-	-	-	-	[73]

Lactobacillus acidophilus NCFM	AAV42075.1	N-acetylmuramidase	4	9.69	6.52	9.85	9.28	10.33	-	-	[73]
Lactobacillus acidophilus NCFM	AAV42379.1	Putative surface exclusion protein	2	9.60	10.39	5.98	-	-	-	-	[73]
Lactobacillus acidophilus NCFM	AAV42924.1	Putative cell surface protein	1	10.08	10.08	-	-	-	-	-	[73]
Lactobacillus acidophilus NCFM	P35829.1	S-layer protein	4	9.59	4.65	8.94	9.43	10.13	-	-	[73]
Lactobacillus acidophilus NCFM	AAV43043.1	Putative enterolysin A	1	9.69	9.69	-	-	-	-	-	[73]
Lactobacillus acidophilus NCFM	AAV43473.1	Putative membrane protein	1	10.02	10.02	-	-	-	-	-	[73]
Lactobacillus acidophilus NCFM	AAV43061.1	Hypothetical protein	1	9.84	9.84	-	-	-	-	-	[73]
Lactobacillus acidophilus NCFM	AAV43453.1	Glycerol-3-phosphate ABC transporter	1	9.73	9.73	-	-	-	-	-	[73]
Lactobacillus acidophilus NCFM	AAV43404.1	PrtM precursor	1	9.79	9.79	-	-	-	-	-	[73]
Lactobacillus acidophilus NCFM	AAV42465.1	Glycerol-3-phosphate ABC transporter	1	9.89	9.89	-	-	-	-	-	[73]
Lactobacillus acidophilus NCFM	AAV43385.1	Putative surface protein	2	9.90	10.06	8.87	-	-	-	-	[73]
Lactobacillus acidophilus NCFM	AAV42253.1	ABC transporter	1	9.98	9.98	-	-	-	-	-	[73]
Lactobacillus acidophilus NCFM	YP_193101.1	S-layer protein	4	9.59	4.65	8.94	9.43	10.13	-	-	[74]
Lactobacillus acidophilus NCFM	AAV42087.1	Putative fibronectin domain	4	9.59	10.12	9.22	8.89	8.19	-	-	[75]
Lactobacillus amylovorus	F0TJ46	S-layer protein X (SlpX)	4	9.50	6.49	6.28	8.96	9.83		-	[76]
Lactobacillus amylovorus	D5GZ50	Putative uncharacterized protein	2	10.56	10.17	11.48	-	-	-	-	[76]
Lactobacillus amylovorus	F2M1V1	S-ayer protein	5	9.66	10.04	6.39	5.79	8.97	9.58	-	[76]
Lactobacillus amylovorus	E4SLF0	Putative uncharacterized protein	2	9.82	9.77	9.79	-	-	-	-	[76]
Lactobacillus amylovorus	E4SJM6	Putative uncharacterized protein	3	10.01	10.39	9.70	9.52	-	-	-	[76]
Lactobacillus amylovorus	F0TE79	Uncharacterized protein	2	9.09	9.23	8.53	-	-	-	-	[76]
Lactobacillus amylovorus	F2M168	Cell separation protein	5	9.82	10.42	9.89	9.27	6.37	6.09	-	[76]
Lactobacillus amylovorus	E4SKI2	Cell separation protein	5	9.82	10.4	9.89	9.05	9.57	5.01	-	[76]
Lactobacillus amylovorus	J4BTR2	S-layer protein	4	9.62	9.98	8.05	5.34	9.40	-	-	[76]
Lactobacillus amylovorus	F0TGG1	S-layer protein	4	9.67	10.11	5.20	5.34	9.48	-	-	[76]

Lactobacillus amylovorus	E4SLY7	S-layer protein	4	9.69	10.11	6.38	5.08	9.56	-	-	[76]
Lactobacillus amylovorus	A8YWK5	Putative uncharacterized protein	3	10.12	10.39	9.92	9.70	-	-	-	[76]
Lactobacillus amylovorus	R5YRQ3	Uncharacterized protein	2	10.36	10.26	10.36	-	-	-	-	[76]
Lactobacillus crispatus MH315	BAC76686.1	Surface layer protein	4	9.46	6.47	8.14	8.83	10.09	-	-	[74]
Lactobacillus crispatus	D5H0Y7	Cell separation protein (CdpA)	4	9.69	10.04	10.09	9.64	4.97	-	-	[76]
Lactobacillus crispatus	D4FH50	Uncharacterized protein	6	9.73	10.09	10.09	9.70	9.70	9.06	4.71	[76]
Lactobacillus crispatus	D0DIV9	Uncharacterized protein	6	9.74	10.09	10.00	9.70	9.46	9.10	4.82	[76]
Lactobacillus crispatus	D5H1S0	S-layer protein X (SlpX)	4	9.68	6.36	5.93	9.57	9.85	-	-	[76]
Lactobacillus crispatus	C2KBQ5	Bacterial group 3 lg-like protein	2	9.09	9.34	8.65	-	-	-	-	[76]
Lactobacillus crispatus	F0NVR1	S-layer protein X (SlpX)	5	9.64	9.05	5.95	9.22	7.92	10.32	-	[76]
Lactobacillus crispatus	D5H0C4	S-layer protein	3	9.55	9.80	9.19	8.73	-	-	-	[76]
Lactobacillus crispatus	C2KEL5	Uncharacterized protein	4	9.41	7.85	10.25	5.03	9.27	-	-	[76]
Lactobacillus crispatus	C2KDQ6	Uncharacterized protein	5	9.69	10.04	10.00	9.78	9.46	5.09	-	[76]
Lactobacillus crispatus	C7XJ17	Uncharacterized protein	3	9.56	10.47	8.90	7.02	-	-	-	[76]
Lactobacillus crispatus	K1M441	Uncharacterized protein	3	9.72	10.47	9.60	8.76	-	-	-	[76]
Lactobacillus crispatus JV-V01	C2KBR4	Bacterial group 3 lg-like protein	2	9.84	9.96	9.54	-	-	-	-	[76]
Lactobacillus crispatus	D4FCG0	Bacterial group 3 lg-like protein	2	9.81	9.88	9.60	-	-	-	-	[76]
Lactobacillus crispatus	D5GYT4	S-layer protein	3	9.85	9.82	10.07	8.15	-	-	-	[76]
Lactobacillus crispatus	C2EM32	Bacterial group 3 lg-like protein	1	9.17	9.17	-	-	-	-	-	[76]
Lactobacillus crispatus	C2KDQ5	Uncharacterized protein	2	10.23	10.16	10.26	-	-	-	-	[76]
Lactobacillus crispatus	U2HNM4	Uncharacterized protein	2	9.92	9.13	10.45	-	-	-	-	[76]
Lactobacillus crispatus	AAB58734.1	Surface layer protein (CbsA)	3	9.69	5.28	8.22	10.02	-	-	-	[79]
Lactobacillus helveticus R0052	AAZ99044.1	Slp precursor, partial	4	9.34	7.71	4.62	9.22	10.24	-	-	[74]
Lactobacillus helveticus JCM1003	BAB72066.1	Surface layer protein	4	9.33	5.44	4.85	8.90	10.12	-	-	[74]
Lactobacillus helveticus	BAB72065.1	Surface layer protein	4	9.21	5.32	4.79	8.90	10.00	-	-	[74]

GCL1001											
Lactobacillus helveticus CP 790	BAA86287.1	Proteinase	4	9.30	5.32	5.00	9.23	10.00	-	-	[74]
Lactobacillus helveticus DPC 4571	YP_001576798.1	Surface layer protein	4	9.77	8.94	4.41	9.70	10.74	-	-	[74]
Lactobacillus helveticus	ADK74769.1	Surface layer protein	3	9.05	6.19	4.62	9.76	-	-	-	[74]
Lactobacillus helveticus CNRZ 892	P38059.2	Surface layer protein	4	9.35	4.45	8.87	8.16	10.17	-	-	[74]
Lactobacillus helveticus	C9M2I1	Bacterial group 3 lg-like protein	1	9.32	9.32	-	-	-	-	-	[76]
Lactobacillus helveticus	A8YUE0	Putative uncharacterized protein	3	9.40	9.92	8.76	8.86	-	-	-	[76]
Lactobacillus helveticus	S5DTT0	Surface layer protein	4	9.54	4.98	8.16	8.93	10.21	-	-	[76]
Lactobacillus helveticus	A8YX60	Putative uncharacterized protein	3	10.04	10.30	9.91	9.55	-	-	-	[76]
Lactobacillus helveticus	J4BP74	Cell separation protein	2	10.20	10.27	10.05	-	-	-	-	[76]
Lactobacillus helveticus	F3MP10	Cell separation protein	2	9.80	9.96	5.38	-	-	-	-	[76]
Lactobacillus helveticus	J3ZCG6	Cell separation protein	4	9.79	10.28	9.73	8.21	5.37	-	-	[76]
Lactobacillus helveticus	A8YWK2	Putative surface layer protein	2	9.92	9.92	9.78	-	-	-	-	[76]
Lactobacillus helveticus	I7JYF0	Surface layer protein	4	9.64	8.97	10.40	8.44	8.82	-	-	[76]
Lactobacillus helveticus	C0XKV9	Uncharacterized protein	1	10.39	10.39	-	-	-	-	-	[76]
Lactobacillus helveticus	J7LF58	Uncharacterized protein	1	10.40	10.40	-	-	-	-	-	[76]
Lactobacillus helveticus	A8YWG0	Putative surface protein	3	9.95	10.38	9.95	6.10	-	-	-	[76]
Lactobacillus brevis	G1UE81	Surface layer protein A	4	9.54	9.96	9.33	6.14	4.90	-	-	[77]
Lactobacillus brevis	Q8GFE5	Surface layer protein B	3	9.54	10.17	8.89	6.32	-	-	-	[77]
Lactobacillus brevis	Q8GFE4	Surface layer protein C	4	9.66	10.32	9.36	9.26	5.18	-	-	[77]
Lactobacillus brevis	Q8GFE3	Surface layer protein D	3	9.68	10.24	8.80	8.33	-	-	-	[77]
Lactobacillus brevis	H9BQS1	Surface layer protein M	4	9.53	10.08	9.19	6.05	6.03	-	-	[77]
Lactobacillus brevis	A0A1W6N798	S-layer protein	4	9.60	10.03	9.58	8.23	5.20	-	-	[77]
Lactobacillus brevis	A0A856KME7	S-layer protein	4	9.68	10.03	9.07	9.300	9.10	-	-	[77]
Lactobacillus brevis	A0A1W6NKR1	S-layer protein	4	9.48	9.93	9.27	4.91	6.06	-	-	[77]
Lactobacillus brevis	A0A856KHL8	Uncharacterized protein	4	9.56	10.03	9.58	8.23	4.93	-	-	[77]

Lactobacillus brevis	A0A1W6NJ64	Uncharacterized protein	4	9.65	10.32	9.52	9.06	4.69	-	-	[77]
Lactobacillus brevis ATCC 367	Q03P39	S-layer proteins	3	9.65	10.17	8.82	8.04	-	-	-	[78]
Lactobacillus kitasatonis	WP_025014788.1	Fibronectin type III domain-containing protein	4	9.64	10.29	9.01	8.14	9.26	-	-	[79]
**Gram negative**											
Bacteroides fragilis NCTC 9343	BF9343_0018	TonB-dependent transporter	3	9.24	4.92	9.57	9.22	-	-	-	[80]
Bacteroides fragilis NCTC 9343	BF9343_4229	TonB-dependent transporter	2	9.02	5.00	9.19	-	-	-	-	[80]
Bacteroides fragilis NCTC 9343	BF9343_1221	OmpA homolog	3	9.45	9.62	5.35	9.35	-	-	-	[80]
Prevotella intermedia 17	PINA0975	Putative TonB dependent outer membrane receptor	1	9.12	-	-	-	-	-	-	[81]
Prevotella intermedia 17	PINA1480	Radical SAM domain protein	1	9.29	9.29	-	-	-	-	-	[81]
Prevotella intermedia 17	PINA0414	Outer membrane lipoprotein	1	9.38	9.38	-	-	-	-	-	[81]
Prevotella intermedia 17	PIN0009	Putative outer membrane lipoprotein	1	9.35	9.35	-	-	-	-	-	[81]

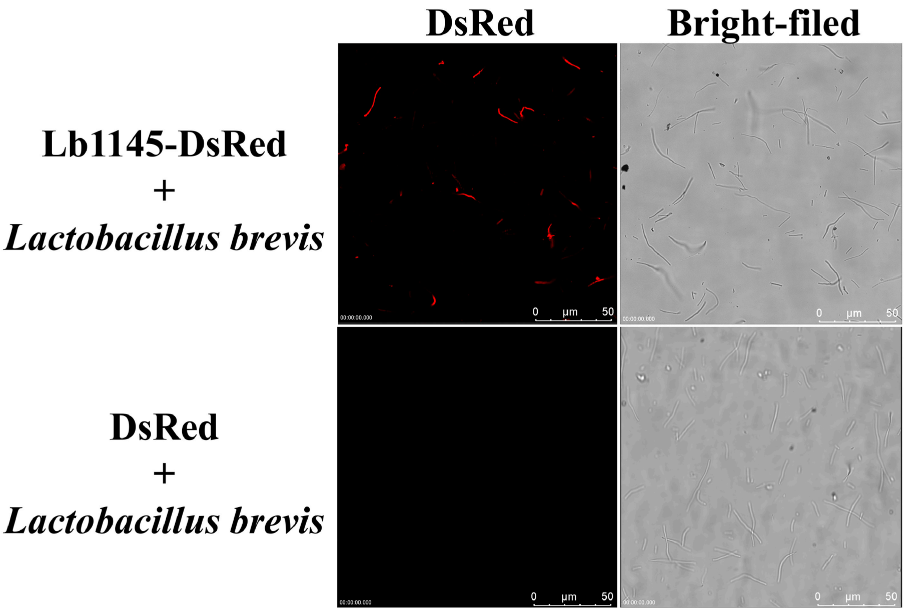

Supplementary Figure S1. Analysis of the binding of the Lb1145-DsRed fusion protein to *L. brevis* as observed with a fluorescence microscope Lb1145-DsRed + *Lactobacillus brevis*: Binding of the fusion protein to *L. brevis*; DsRed + *Lactobacillus brevis*: Binding of the DsRed control to *L. brevis*.
